# Know the Risk: Stroke With Patent Foramen Ovale

**DOI:** 10.7759/cureus.47447

**Published:** 2023-10-22

**Authors:** Rebekah Lantz, Sydney N Carnes

**Affiliations:** 1 Internal Medicine, Miami Valley Hospital, Dayton, USA; 2 General Medicine, Wright State Boonshoft School of Medicine, Dayton, USA

**Keywords:** neurologic deficits, paradoxical emboli, cva, pfo closure, dapt, mca, nih, stroke distribution, stroke, pfo

## Abstract

The presence of patent foramen ovale (PFO) is noted to be higher in patients with a history of cryptogenic stroke, especially in younger patients <55 years old. PFO has shown to be a relatively common occurrence in the population, in 25-30% of individuals. Our case is one of right middle cerebral artery (MCA) infarct due to thromboembolism from a PFO. A 44-year-old white right-handed woman with a history of insulin-dependent diabetes mellitus, hyperlipidemia, hypertension, tobacco abuse, and obesity presented with complaints of new onset headache, dizziness, and left arm and leg heaviness, suspicious for right MCA lesion. She was admitted with stroke-like symptoms, National Institute of Health Stroke Scale (NIHSS) of 8 for left-sided weakness, sensory loss, and ataxia. Computed tomography (CT) head was negative for hemorrhage, and there was no large vessel occlusion on computed tomography angiogram (CTA). She was aspirin-loaded and started on dual antiplatelet therapy (DAPT). Ultimately, brain MRI showed right MCA ischemic stroke, and full stroke assessment showed small PFO on the transthoracic echocardiogram (TTE). She was continued on aspirin and clopidogrel DAPT for 21 days, followed by aspirin monotherapy. Unfortunately, her left-sided deficits did not completely resolve, and she was discharged to rehab. She has had recurrent stroke and is currently considered for PFO repair. A patient's past medical history, last known well time, and exacting symptoms with the NIHSS at onset should be thoroughly obtained at the first medical contact. CT imaging should rule out hemorrhage prior to prompt antiplatelet or thrombolytic administration. In addition, when there are absence of risk factors and the cause remains unknown, it is especially important to obtain TTE with Doppler to assess for right-to-left atrial shunt indicating PFO and potentially contributing thromboembolic etiology. Stroke precautions involving swallow evaluation, aspiration and fall precautions, serial NIH for changes, sequence of imaging, and physical therapy (PT) and occupational therapy (OT) should entail. A stroke neurologist should also be involved at presentation, with the stroke alert protocol shown to improve patient outcomes. Additional risk factors, such as PFO, should also be addressed, often with a multimodal team of providers and careful weight given to the risks and benefits of invasive procedure.

## Introduction

Strokes are cerebrovascular events that oftentimes lead to debility. Defects can improve but may be permanent, and the variety of expression depends on the neurologic distribution [1-2]. Patients must be fully assessed for the level of consciousness, response to commands, vision loss, facial asymmetry, weakness and motor drift, gait abnormality, sensory losses, dysarthria and aphasia, and awareness of extremities in space. These describe the National Institute of Health Stroke Scale (NIHSS) [3]. In addition to the NIHSS, additional cognitive tests, such as the Montreal Cognitive Assessment (MoCA) tool, can assess for further visuospatial, executive, working memory, language, and orientation defects [4]. Transient ischemic attacks (TIAs) are similar events that do not show up on brain imaging and have a resolution of symptoms. They are treated and managed similarly with stroke, with a comparable level of lifetime risk [5]. The umbrella term for TIA and stroke is cerebrovascular accident (CVA).

CVAs can be one or several of the categories, namely, ischemic, hemorrhagic, or subarachnoid [2]. The ischemic type can be thrombotic, such as from inflammation, plaque buildup, or intrinsic vascular dysfunction. Ischemia can also result from an embolic cause, from a local plaque that thromboses or from a cardiac thrombus in an arrhythmic event [2]. The metabolic syndrome risk factors including hypertension, hyperlipidemia, diabetes mellitus, and obesity contribute to the risk as does arrhythmia [6]. CVAs can also be cryptogenic or unknown [7]. A full stroke workup per the guidelines helps to delineate these risk factors, and it has been shown that order sets within the electronic medical record (EMR) of use helps to eliminate the risk of missing any of these components [8]. The assessment starts with the presenting concern, timing, and assessing the distribution of symptoms. A rapid computed tomography (CT) rules out the hemorrhagic cause, and the other types of CVAs can safely be worked up [2]. CT angiography (CTA) of the head and neck establishes if there is a large vessel occlusion where endovascular intervention would be of use and further delineation of candidacy ensues [2]. A complete workup should entail a stroke center of excellence, ideally with an on-site neurology team, but this is not always possible in remote areas of the country and patient care should otherwise be optimized [9]. A transthoracic echocardiogram (TTE) with Doppler and bubbles assesses for an embryologic lack of closure in the cardiac membrane between the top two chambers of the heart or atria [10]. When a hole is present within this membrane, it is termed patent foramen ovale (PFO), which is a contributing etiology to CVA, as in our case.

PFO is a type of cardioembolic risk factor that contributes to 25% of stroke cases in patients who have a right-to-left atrial shunt [10]. The size and prevalence of PFO are similar for males and females, and increased age at diagnosis is associated with a larger size of PFO. In patients where the etiology of stroke is unknown or cryptogenic, PFO appears to be a plausible cause [11]. Paradoxical embolism from venous circulation can occur when thrombus formation along the PFO embolizes and exits the heart to the brain [10]. Thus, patients with known PFO should be on appropriate antiplatelet therapy, and anticoagulation should be considered. The size and level of contribution of PFO certainly involve complex medical decision-making, and a multidisciplinary team of providers as operative closure does not come without risks.

Here, we discuss the case of a middle-aged woman with a diagnosis of ischemic right middle cerebral artery (MCA) stroke, where workup showed a thrombus from preexisting PFO. We also discuss the deficits associated with different regions of stroke and, as for our patient, the potential long-term neurologic deficits associated therein.

## Case presentation

A 44-year-old right-handed Caucasian woman had a history of insulin-dependent diabetes mellitus, hyperlipidemia, hypertension, morbid obesity, tobacco abuse, obstructive sleep apnea, and chronic pain. She was moderately active, had no personal or familial clotting history, and was not recently or currently pregnant. She presented to the emergency department (ED) in February 2023 with complaints of new onset headache, dizziness, and left arm and leg heaviness, suspicious for right MCA lesion. Her last known well was six hours prior to presentation.

She was hemodynamically stable with a temperature of 97.7 Fahrenheit, pulse 90 beats per minute, respirations 18 per minute, blood pressure 134/61 mmHg, oxygen saturation 98% on room air, and body mass index (BMI) 41.6 kg/m^2^. She was oriented to four domains and her memory and concentration intact. Face was symmetric, and she had clear speech without dysarthria. Visual fields were intact in all quadrants, and there were no conjugation defects or nystagmus. Strength was 5/5 to right-sided upper and lower extremities, but her left upper extremity was 0/5. It was unclear if this was simply due to effort. She endorsed decreased sensation to the left upper and lower extremities in addition to the motor deficits mentioned, and sensation diminished V1-V3 to the left face. Deep tendon reflexes were normal throughout, and there was no clonus or Babinski signs.

**Figure 1:**
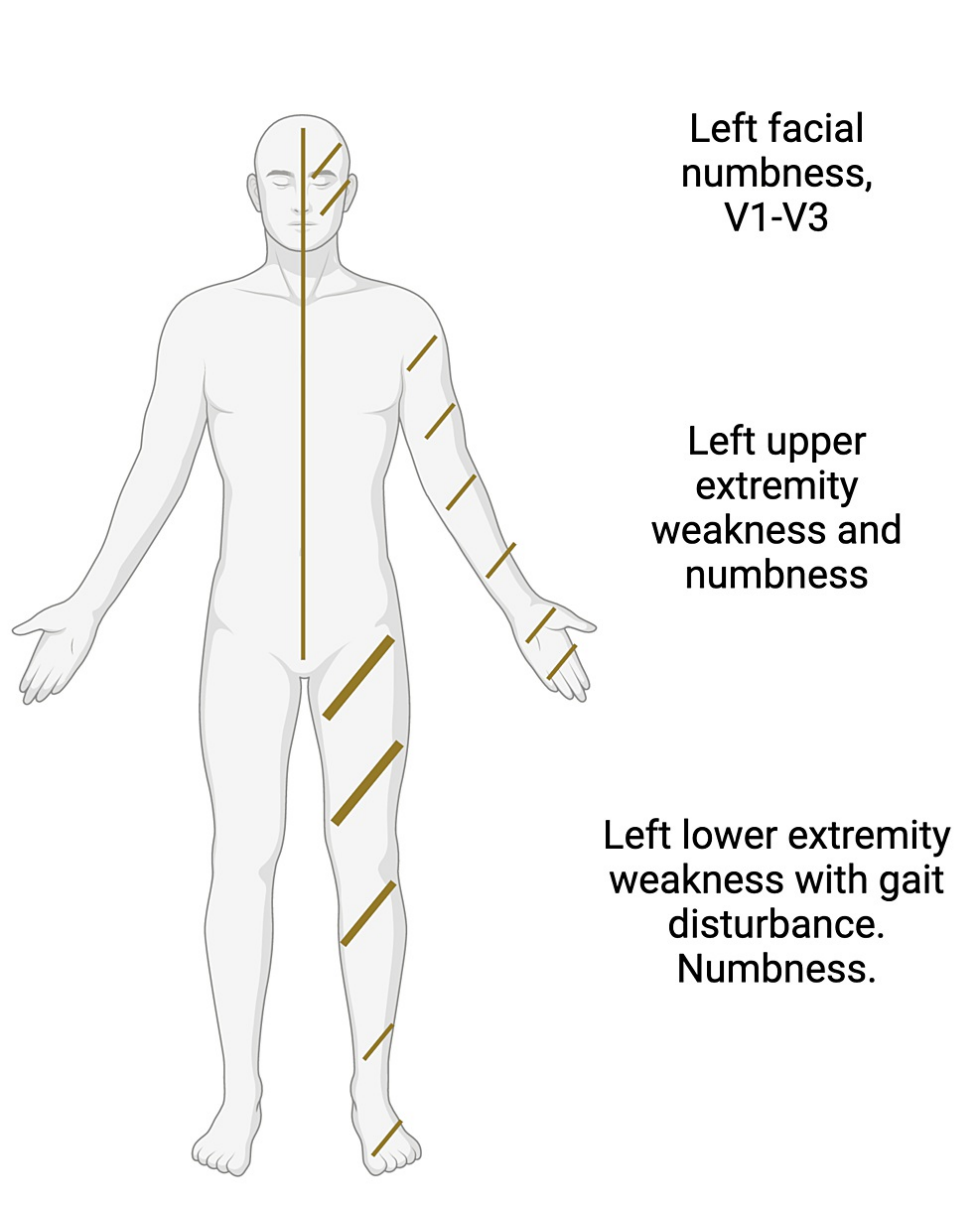
Index neurologic deficits Created with BioRender.com.

The NIHSS was 8 for left-sided weakness, sensory loss, and ataxia. This was similar on the ED provider and with the neurology telemedicine encounter. Pertinent positives are noted on Figure 1.

**Figure 2:**
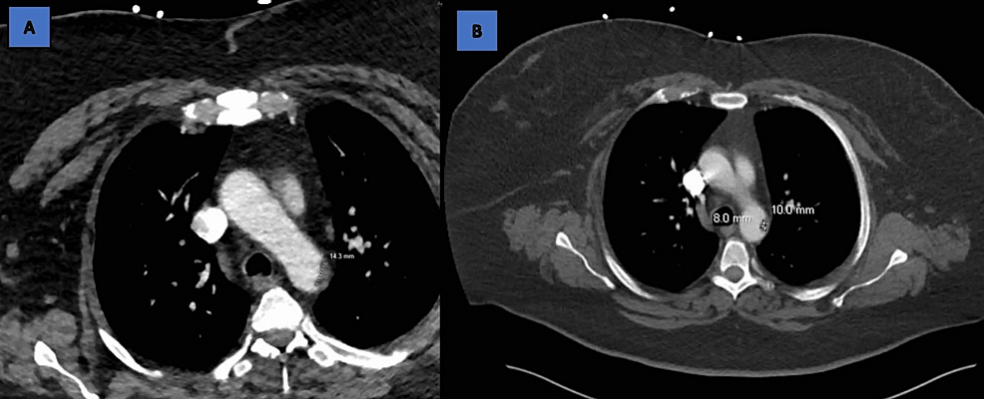
A. CTA head and neck shows filling defect at the aortic isthmus measuring 1.4 cm, unchanged from the previous. Consider focal noncalcified plaque or mural thrombus. B. CT aortic dissection protocol. The prior imaging in January 2023 that demonstrates focal distal aortic arch/proximal descending aortic plaque 1.0x0.8x1.1 cm. CTA: computed tomography angiogram

Admission labs were consistent with hyperglycemia 207 mg/dL but otherwise normal on the chemical and blood count panels. Initial CT head without contrast was non-acute and showed no hemorrhage or overt ischemic changes. CTA head and neck showed no large vessel occlusion (LVO), but it did capture thoracic thrombus in ascending thoracic aorta (Figure 2A). Of note, this was a stable finding from a CT aortic dissection protocol in January or a filling defect from contrast load (Figure 2B). Tissue plasminogen activator (tPA) and tenecteplase (TNK) were contraindicated due to the presentation time >four hours from onset of symptoms and stable NIHSS, and an endovascular therapy was warranted given the absence of LVO. However, due to the apparent thrombus, hypercoagulable labs were collected (Table 1). She was started on heparin drip, admitted to the hospitalist, and consulted under cardiology.

**Table 1:** Hypercoagulable lab results Definitions: ACT, activation of plasminogen to plasmin; APL,  IgA phosphatidylserine units; APS, IgA phospholipid units; APTT, activated partial thromboplastin time; DRVVT, diluted Russell viper venom time; GPL, IgG phosphatidylserine units; GPS, IgG phospholipid units; Ig, immunoglobulin; INR, international normalized ratio; LA, lupus anticoagulant; LMWH, low-molecular-weight heparin; MPL, IgM phosphatidylserine units; MPS, IgM phospholipid units; N/A, not applicable

Lab test	Patient result	Reference value
DRVVT screen	55.6	30.1-44.1 sec
Koalin clotting time	101.1	53.1-86.0 sec
Platelet count	253	140-400 K/uL
Total homocysteine	10.8	4.9-14.8 mcmol/L
Prothrombin gene mutation	Not detected	Not detected
Anti-thrombin III	100	80-120%
Protein C activity	122	70-130%
Plasminogen ACT	109	80-120%
Activated protein C resistance	2.1	>2.0 ratio
Free protein S	117	50-150%
Total protein S	83	60-150%
IgA, beta 2 Glyco	12	<20 A units
IgG, beta 2 Glyco	4	<20 G units
IgM, beta 2 Glyco	47	<20 M units
Anti-prothrombin IgG	2	<20 G units
Anticardiolipin, IgA	3	<22 APL
Anticardiolipin, IgG	6	<23 GPL
Anticardiolipin, IgM	26	<11 MPL
Phosphatidylserine IgA	0	<20 APS
Phosphatidylserine IgG	2	<16 GPS
Phosphatidylserine IgM	20	<22 MPS
APTT LA screen	42.0	30.8-44.8 sec
Thrombin time	18.0	15.0-20.0 sec
Prothrombin time	13.1	11.7-13.9 sec
INR	1.0	0.9-1.1
APTT	32.3	24.5-35.2 sec
Pathologist Interpretation	Patient is on LMWH	N/A

**Figure 3:**
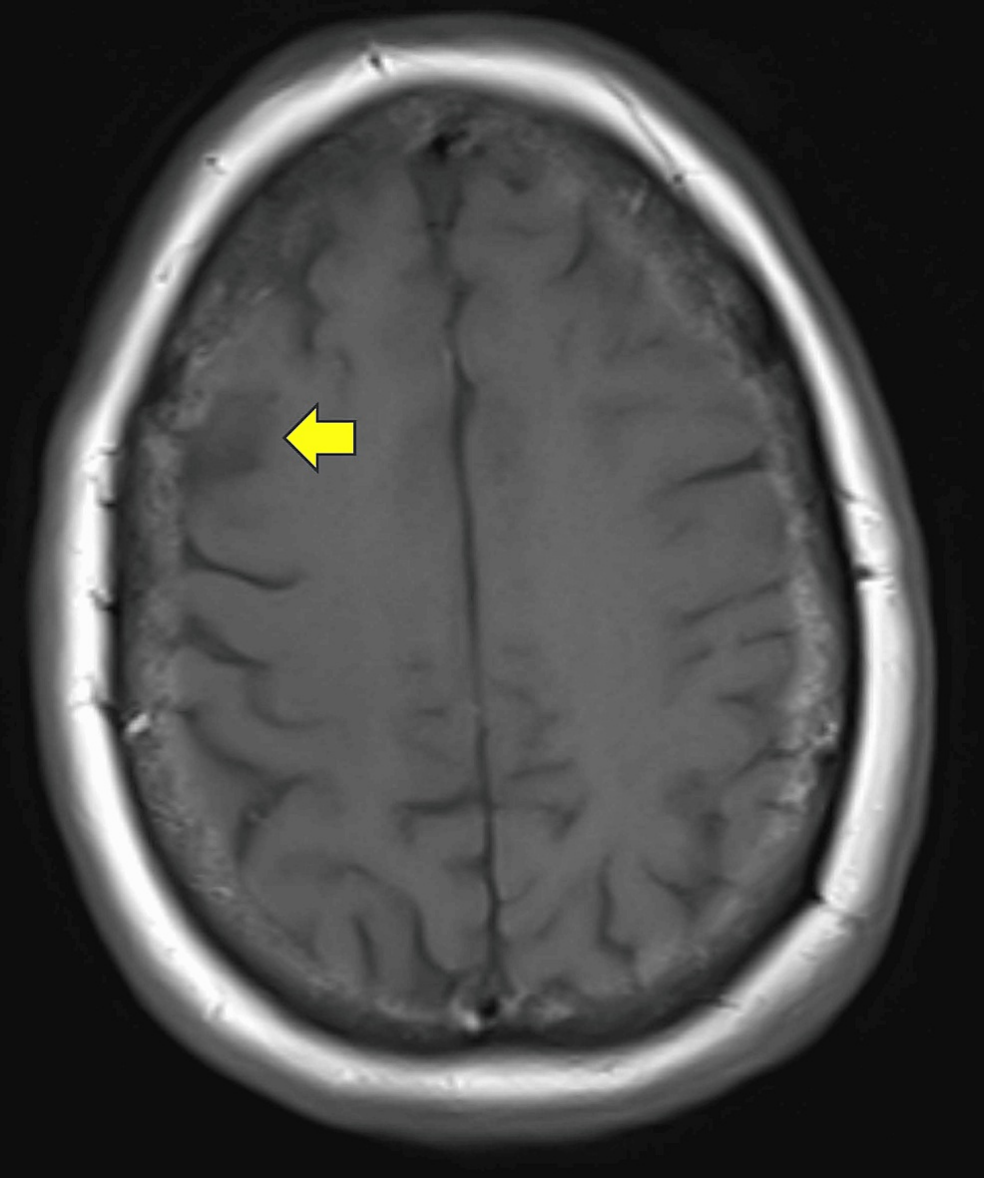
MRI brain without contrast shows subacute ischemia in a right MCA distribution without intracranial hemorrhage Definitions: MRI, magnetic resonance imaging; MCA, middle cerebral artery

A TTE was consistent with the thrombus of the ascending aorta distal to the aortic arch, and she had a positive bubble study, indicating the presence of an intracardiac intra-atrial shunt, essentially a PFO. Ultimately, MRI brain without contrast showed right MCA distribution cortical subacute ischemia without hemorrhagic transformation (Figure 3). Hypercoagulable labs were largely unremarkable. The ischemic stroke was considered unrelated to the thrombus due to the location and was therefore thought to be an incidental finding. PFO was small and there was no indication for closure and no indication for long-term anticoagulation, and an outpatient mobile heart monitor was ordered.

Unfortunately, the patient continued to have left hemiplegia and difficulty with ambulation. After physical and occupational therapy assessments, it was determined that she would benefit from inpatient rehab. At time of discharge, she was optimized on anti-lipid therapy with high-intensity atorvastatin 80 mg and fenofibrate 145 mg, which were new medications for her. DAPT with aspirin 81 mg and clopidogrel 75 mg were continued for 21 days, followed by aspirin monotherapy. She was given return precautions and scheduled for outpatient stroke follow-up.

**Figure 4:**
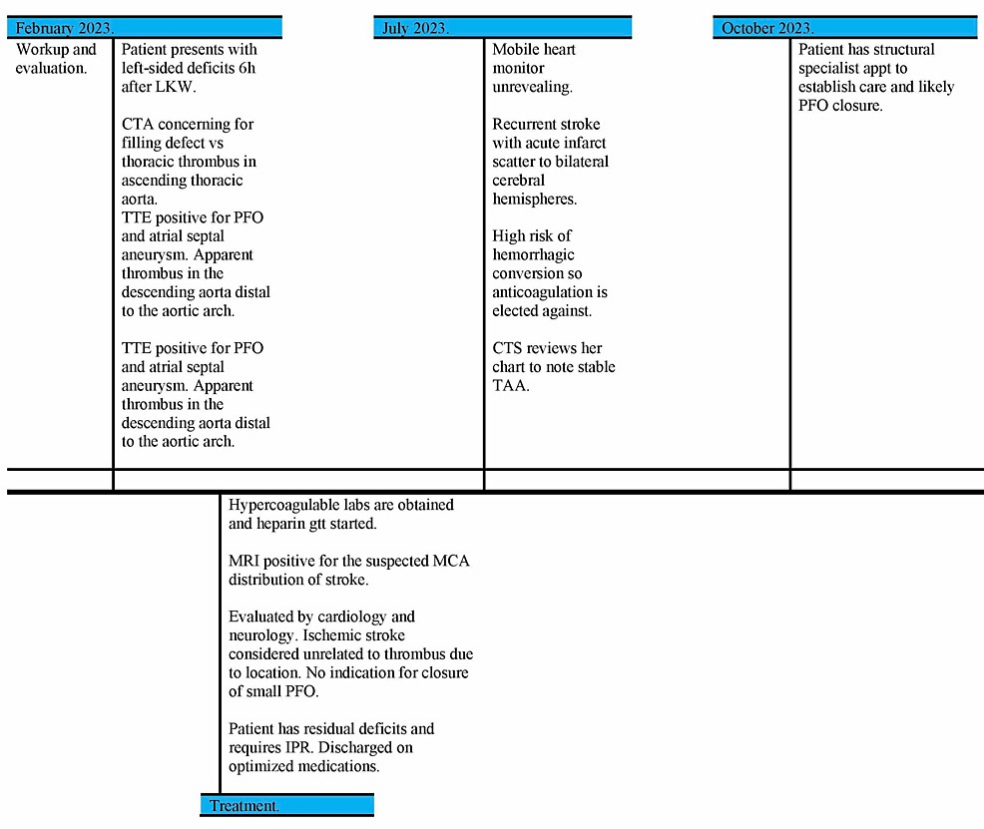
Timeline of the patient’s chronologic course Definitions: appt, appointment; CTS, cardiothoracic surgery; IPR, inpatient rehab; LKW, last known well; MCA, middle cerebral artery; MRI, magnetic resonance imaging; PFO, patent foramen ovale; TAA, thoracic aortic aneurysm; TTE, transthoracic echocardiogram

She has since been admitted and discharged for workup of recurrent stroke in July of the same year, with acute infarct scatter to bilateral cerebral hemispheres. Results of the heart monitor were noncontributory, but she was noted to have a 2.4 second nocturnal pause. More due to recurrence than to an arrhythmic etiology, the PFO became more concerning with contribution to recurrent cardioembolic strokes. She was to have follow-up imaging and meet with the structural heart specialist. Therapeutic anticoagulation was deferred due to her high risk of hemorrhagic conversion. Her CTA chest in August was stable, and she will see structural cardiology to establish care and schedule for a likely PFO closure in October of the same year. Figure 4 details the patient’s chronological timeline.

## Discussion

As noted in our case of ischemic and likely secondary cardioembolic stroke related to PFO, CVA events can lead to permanent debility, high healthcare costs, morbidity, and mortality [1,12]. In the index presentation, the patient experienced an MCA distribution neurologic deficit. Cortical infarcts are largely composed of three main cerebral categories: anterior (ACA), middle (MCA), and posterior cerebral artery (PCA). There are also less common distributions of stroke, namely, vertebrobasilar, cerebellar, and lacunar, but the MCA lesion as a branch from the internal carotid artery (ICA) remains the most prevalent involved vessel. This is likely due to availability; it is the largest vessel, and therefore its distributions are more likely to be affected [2]. Vasculature distribution is important to consider and can sometimes be predicted from symptoms even before MRI results.

The MCA, as in our case, is the most common artery involved in stroke. It typically presents with contralateral upper extremity flaccidity and ipsilateral gaze defect. Phonation dysarthria, aphasia, and hemineglect, along with contralateral sensory deficits, may present [2].

ACA strokes alone are uncommon due to collateral blood flow that compensates for ischemic changes [2]. A patient would likely present with a mixed distribution. The ACA supplies the midline frontal lobe, medial and superior portions of the parietal lobe, and Broca’s region [2]. Therefore, deficits involve contralateral lower extremity weakness and sensory changes of the leg and foot, personality changes, agitation, memory deficits, and difficulty with speech initiation [2,13].

The PCA is a branch of the basilar artery, which is formed from the fusion of the vertebral arteries. This vessel supplies the occipital lobe, inferomedial temporal lobe, a majority of the thalamus, and portions of the brain stem [2]. PCA strokes involve the visual fields due to supply of the occipital lobe, so patients often report vision loss and classically contralateral homonymous hemianopsia with macular sparing. However, diplopia and difficulty recognizing colors and faces may present. When deeper structures are involved, patients would experience hemisensory loss and hemiparesis with the involvement of the internal capsule. Specifically, proprioception, tactile sensation, and stereognosis are affected. Amnesia and headache can sometimes occur [2].

**Figure 5:**
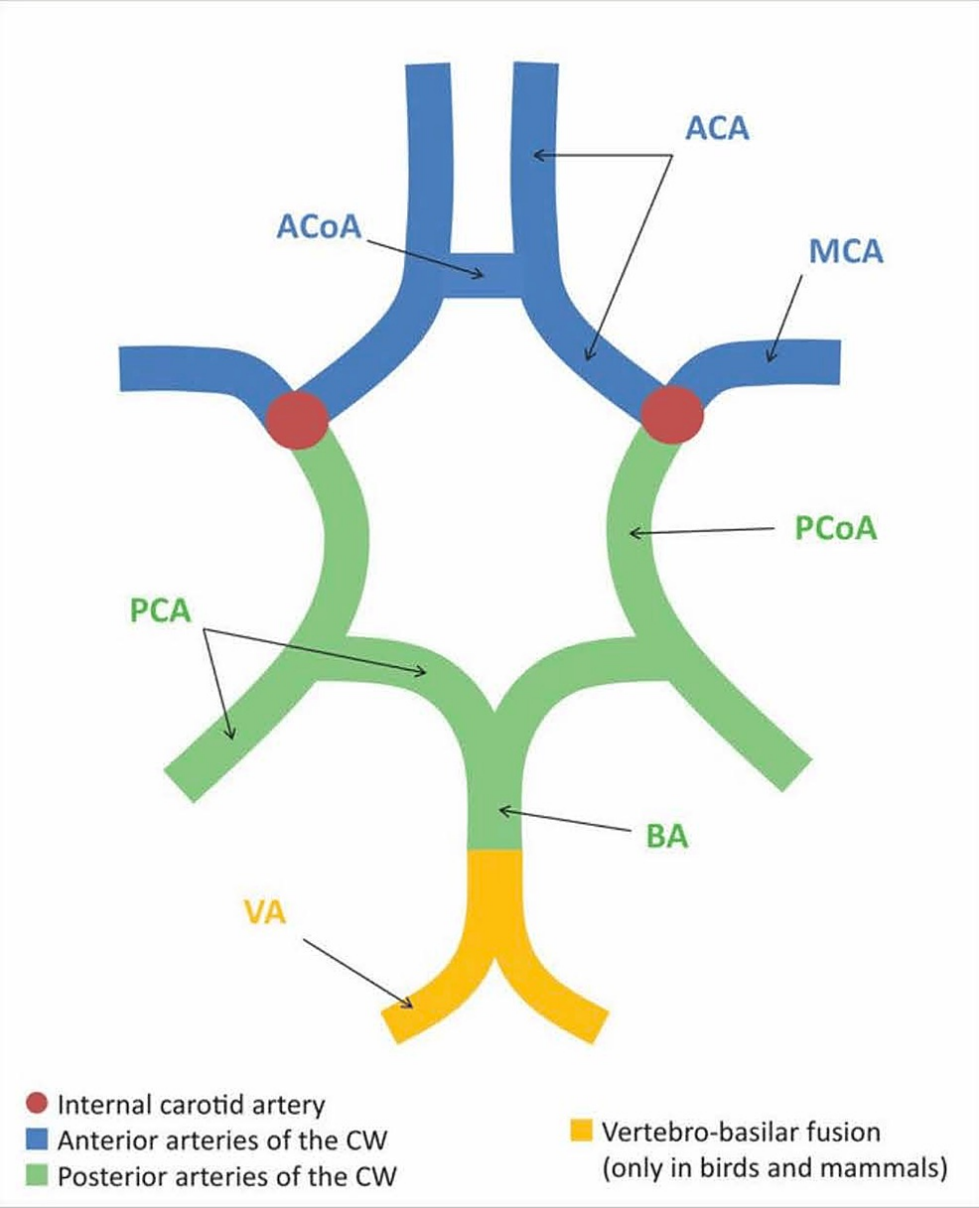
Circle of Willis Definitions: ACA, anterior cerebral arteries; ACoA, anterior communicating artery; BA, basilar artery; CW, circle of Willis; MCA, middle cerebral artery; PCA, posterior cerebral arteries; PCoA, posterior communicating artery; VA, vertebral artery. Image is unchanged and shared from [14] under the CC BY license.

Vertebrobasilar stroke can best be demonstrated by the circle of Willis (Figure 5), where the blood supply arises from the vertebral and basilar arteries. Given that cerebellar and brain stem structures are vascularized, one would expect to see ataxia, vertigo, and headache. Sometimes, visual field defects, emesis, and tongue and pharynx deficits occur [2].

Cerebellar stroke is very similar to that of vertebrobasilar, in vascularization and symptomatology. In addition, cerebral edema can occur. These strokes are especially dangerous given the rapid clinical deterioration that is associated with this distribution [2].

Lacunar stroke involves the small perforating arteries in the deep structures of the brain. Because of the small size, local intrinsic occlusion or embolism is typically suspected. An event may occur as a silent stroke or it may cause pure somatic or sensory losses. Sometimes, the effect is larger and leads to ataxia or hemiparesis [2].

When evaluating patients with stroke-like symptoms, it is important to obtain a careful history including a last known well time, exam including an accurate NIHSS, and imaging including stat CT to rule out hemorrhage. These should proceed the consideration of tPA/TNK therapies; otherwise, patients should be aspirin- and/or clopidogrel-loaded per standard of care with neurology and therapy services in consult. It is also important to allow permissive hypertension (<220/110), followed by blood pressure control (<160/90) after 24 hours to prevent intracranial pressure or stroke transformation. Anti-lipid therapy should be added to maximum tolerated doses to slow down plaque progression and glucose, especially for patients with diabetes, and should be tightly controlled for best outcomes. Moreover, full assessment should evaluate for PFO, which is present in 25% of the population and may contribute to stroke with consideration for closure [11]. Our patient had an interesting finding of thrombus that was ultimately thought to be noncontributory versus a contrast filling defect, and therefore no intervention was deemed necessary. A multidisciplinary team of specialists is needed for ideal stroke management.

If PFO is found to contribute to the thromboembolic cause of stroke, as in the presented case, steps can be taken to help reduce further risk and prevent recurrence, such as thrombolysis, anticoagulation, and PFO closure [15-16]. Each of these do not come without risk and should be carefully considered with a multimodal team of specialists. Especially, PFO closure has not universally been deemed the standard of care after several randomized controlled trials [15,17]. Thrombolysis has a mortality rate of 36% [16]. Anticoagulation, typically with heparin drip, has an associated mortality rate of 14%. Surgical closure was reported to have a 13% mortality rate, and this is done after patients are carefully considered for candidacy. A combination of these treatments can also be attempted, considering the individual patient case [16].

Stroke cases are not always straightforward or simple. Our case was complicated by irreversible debility in the index stroke, followed by recurrence in a wider distribution, so risk factors were further reassessed. Ultimately, for our patient, this may lead to more invasive surgical intervention. Conversations should be as clear as possible with patients and families, regarding the candidacy for surgery and the risks associated with procedures. It is well known that stroke begets stroke; that is, after an initial stroke, the risk of consecutive stroke is more likely. Overall, prevention is key, and all physicians should portray knowledge regarding wellness and healthy lifestyle.

## Conclusions

The distribution of stroke can oftentimes be predicted by a patient’s presenting symptoms based on the knowledge of vascular supply and the function of neural regions of the brain and brain stem. It is important to collect information at the first medical contact regarding last known well time and follow with immediate non-contrast head CT to depict candidacy for thrombolysis or neurosurgical intervention. A standardized stroke protocol helps to guide inpatient and post-stroke care. Meanwhile, some strokes are simple workups, not all are, as for our patient with visualized thoracic thrombus and PFO on TTE. To continue carefully weighing the risks and benefits of various treatment modalities, involving a multidisciplinary team of providers and maintaining clear communication with the patient and family are of utmost importance for best outcomes. Ultimately, stroke prevention is key, and we encourage healthcare providers to be strong proponents for lifestyle changes that lead to overall better quality of life.
